# Voices from the foodbank: experiencing food insecurity in North-West England during the cost-of-living crisis

**DOI:** 10.1093/pubmed/fdag036

**Published:** 2026-05-18

**Authors:** Koser Khan, Natalija Atas, Daniel Clarkson, Linda Heaton, Becky Shaw, Adele Thomas

**Affiliations:** Division of Health Research, Faculty of Health and Medicine, Lancaster University, Bailrigg Lane, Lancaster LA1 4YW, UK; School of Social Sciences, Liverpool Hope University, Hope Park, Liverpool, L16 9JD, UK; Division of Health Research, Faculty of Health and Medicine, Lancaster University, Bailrigg Lane, Lancaster LA1 4YW, UK; Division of Health Research, Faculty of Health and Medicine, Lancaster University, Bailrigg Lane, Lancaster LA1 4YW, UK; Applied Research Collaboration Northwest Coast, Institute for Population Health, Brownlow Street, Liverpool University, Liverpool L69 3GL, UK; Applied Research Collaboration Northwest Coast, Institute for Population Health, Brownlow Street, Liverpool University, Liverpool L69 3GL, UK

**Keywords:** mental health, food choice, poverty

## Abstract

**Background:**

The current cost of living crisis in the UK has exacerbated food insecurity and the associated impacts on people’s health and well-being. There is limited understanding of the extent and depth of the health impacts on those facing food insecurity. This qualitative study examines lived experiences of food bank users facing food insecurity in the North-West of England and the impacts on their mental health.

**Methods:**

18 semi-structured interviews were conducted with participants recruited from three food banks in North-West of England. Thematic analysis was used to analyze interview data. Two public advisors also reviewed analysis.

**Results:**

Participants described the challenges of being food insecure while accessing support from a food bank and how this had detrimental impacts on their mental health. Four main themes of mental health impacts emerged from the interviews: precarious circumstances, loss of self-worth and dignity, loss of agency, and parental guilt.

**Conclusions:**

Individuals accessing food banks face severe and detrimental impacts on their mental health and wellbeing. Food bank users commonly reported increased anxiety, stress, and depression, along with feelings of parental guilt and loss of dignity. Consideration of mental health needs is required and how perceptions of shame and stigma can be reduced.

## Introduction

Food insecurity refers to insufficient and inconsistent access to food, which hinders the ability to meet dietary requirements, food preferences, and maintain adequate nutritional intake, ultimately impacting an individual’s health and overall well-being.^1^ Food insecurity is a significant public health concern, as it can lead to physical and mental health problems, contributing to unequal health outcomes across the population.^2–5^

A combination of national and global economic and geopolitical challenges has created an unprecedented set of circumstances that have spiked inflation and triggered the Cost-of-Living Crisis in the UK.^6^ For example, the *United Kingdom Food Security Report 2024* notes that the period from 2021 to 2024 was affected by the COVID-19 pandemic, as well as the development of trade relationships with European economic partnerships following Brexit.^7–9^

The consequent Cost-of-Living Crisis, characterized by an unprecedented rise in electricity, fuel and food costs, has contributed to the increase of food insecurity and caused significant hardship for millions of individuals across the UK.^10^^,^^11^ The crisis also increased inequalities, as households in the most vulnerable communities in the UK have been disproportionately affected with the lowest incomes experiencing a higher-than-average inflation rate and facing a sharp decline in living standards.^6^^,^^12–14^

In 2024, an estimated 7.2 million adults and 2.7 million children in the UK experienced food insecurity, representing ~14% of all households.^15^ Data from DWP also showed that in 2023/24 2.8 million people in the UK lived in households that had support from a food bank in the last 12 months.^16^ During the same period, Trussell food banks distributed ~2.9 million emergency food parcels, marking a 51% increase over the past 5 years.^17^ These figures illustrate the pervasive extent of the issue across the United Kingdom, highlighting the urgent need to implement evidence-based interventions and policy responses.

Although scholarly interest in food insecurity has grown considerably over the past decade,^18–22^ there remains limited understanding of first-hand experiences of food insecurity and its effects on mental health and wellbeing, particularly in the context of the ongoing Cost-of-Living crisis. This qualitative study contributes to addressing this gap by providing nuanced insights into the lived experiences and health consequences of food insecurity among food bank users in the North-West, one of the UK’s most deprived regions.

## Methods

The study was conducted in three areas of North-West England between January and March 2025: a highly deprived urban area, particularly for children, with high unemployment; a majorly deprived urban area, particularly for children and old people, with high unemployment; and an intermediate urban area with lower deprivation and below average unemployment.^23^^,^^24^ Food banks were identified through existing Applied Research Collaboration North-West Coast (ARC NWC) connections with Trussell Lancashire and Cumbria food banks cluster group, and the contacts of those organizations. They were selected based on interest and willingness to be involved in the study.

Field work was conducted by three researchers (KK, DC, LH).18 semi-structured interviews were undertaken. Interview questions explored participants’ experiences of food banks, reasons for accessing support, impacts on life and the health and well-being of participants and their families, and support provision. A qualitative interview design was selected as it allowed a more in-depth exploration of people’s experiences and perceived impacts of using food banks. Qualitative research also gives voice to lived experiences helping share participant narratives with others.

Recruitment was undertaken at three food banks with the support of a designated food bank staff member. Purposeful sampling was used to identify individuals accessing food banks or who had accessed a food bank in the last 3 months. Diversity was sought in the sample. Just over half the sample was female, largely aged between 25 and 44 from a White British background. One quarter identified as an ethnic minority and the same proportion reported a disability. Over a third had a long-term health condition. Most participants were unemployed and were predominately from single-adult households with children.

Participants were approached either face-to-face by the designated member of staff at the food bank or by telephone, provided with an information sheet and asked to sign a consent form before interviews were undertaken. Participants were also asked to complete an anonymized monitoring form requesting basic demographic information (Table 1). Interviews were undertaken in person at food banks, Microsoft Teams video call, or by telephone call according to participants’ preferences. Interviews lasted 30–45 min.

**Table 1:** Participant demographics.

		Number	Percentage
**Gender**	Male	7	39%
	Female	11	61%
**Age group**	25–44	12	75%
	45–64	3	19%
	Over 65	1	6%
**Ethnicity**	Identified as a minority group i.e. not white British or English	4	25%
**Disability**	Identified as having a disability	4	25%
**Long Term Conditions**	Identified as have a long-term chronic health condition	6	38%
**Employment status**	Employed	1	6%
	Not employed	13	81%
	Student	1	6%
	Prefer not to say	1	6%
**Household occupancy**	Single Adult household with children aged 0–16 years	14	88%
	Single Adult household without children	1	6%
	Multi-Adult household with children aged 0–16	1	6%

Sufficiency of data was guided by information power. Participants were specific to the study aim and provided rich accounts. Therefore, consistent themes began to emerge early on in data collection enabling data sufficiency to be reached within the sample. Researchers adopted a reflexive approach throughout the study, considering how their experiences and roles as researchers might influence data collection and interpretation, to minimize bias participant perspectives were kept central. This was supported through regular meetings with the researchers during data interpretation.

Interviews were audio recorded and transcribed. Inductive thematic analysis was undertaken guided by Braun and Clarke’s six phases of thematic analysis, which was utilized as it provides a systematic framework for organizing qualitative data and interpreting meaning within data.^25^ Lumivero NVivo15 qualitative data analysis software was used to conduct data analysis.

A coding framework was developed by researchers, initially coding and discussing one transcript to help define codes as they emerged. Researchers then coded the data. The framework was reviewed and modified—researchers met to discuss coding reliability and theme generation, then reviewed coding to check for consistency and the reliability of themes before reaching agreement on the higher-level themes and sub themes. Two public advisors also reviewed emerging themes to ensure that these were reflective of how those with lived experiences would describe their experiences.

Pseudonym names have been used for quotes, and a numbered area has been used for each participant to demonstrate views across the three research sites.

## Ethics

Ethical approval was granted by Lancaster University Faculty of Health and Medicine ethics committee (FHM-2024-4718-RECR-2) in January 2025.

## Results

A total of 18 participants took part in the study; Table 1 provides key demographic information. Two participants did not provide answers for all information requested. A high proportion of the sample was unemployed (81%), White British; aged between 25 and 44 years old (75%) and were from single adult households with children (88%).

Four major themes affecting participant mental health and wellbeing were identified: (1) Precarious circumstances, (2) Loss of self-worth and dignity, (3) Loss of agency, and (4) Parental guilt and concealed struggles.

### Precarious circumstances

The financial struggles experienced by participants were a major factor impacting mental well-being as well as contributing to declining mental health, which frequently manifested in episodes of severe anxiety and depression. Participants felt immense pressure of making ends meet and not having sufficient money to purchase essentials, often leading to feelings of helplessness.

Everything is linked with having no money, having… Like you know, if you can't afford things… so it is affecting me mentally, it’s affecting my health as well because I’m stressed and depressed (Zainab Area 1)*.*

The uncertainty of where food was going to come from or what they would be able to make to feed their families weighed heavily on participants’ minds.

I’ve got really bad anxiety; I just sit and worry about what I’m gonna make for tea and stuff like that. You know, … What I’m gonna do for breakfast.... Just sometimes I forget that we’ve no butter, and then I realise I can’t make the kids bloody toast for breakfast, but there’s no cereal as well... it really does affect you; I’ve cried numerous times over it (Kylie Area 3).

This often led to feelings of sadness and frustration about the inability to cook and eat what they wanted, as well as increased anxiety levels.

When you don’t get enough like fruit and vegetables you feel tired... you start feeling worn out... With my anxiety and depression… I start feeling like I’m a shit parent, I overthink things, more, …, my brain goes into overdrive then (Sarah Area 3).

Participants often faced difficult decisions, such as having to choose between necessities and utility bills. The uncertainty of their circumstances often led to participants feeling overwhelmed.

‘It’s disturbing a lot, but sometimes you say, oh my God, how can I manage this month? I have to do this; I have to do this (Fatima Area 1).

I suffer with depression... sometimes you feel low, don’t you, if you’ve got no food, you feel a bit anxious cause you’re thinking where I gonna [go], who am I gonna turn to (Heather Area 2).

Not having access to sufficient food was recognized as being one of the key factors in impacting overall mental well-being, while also impacting participants’ overall ability to cope with difficult circumstances.

It’s harder to think through things if you ain’t got food. You can be more depressed, you’re hungry as well, you ain’t got the energy to think… It can add to stress…, thinking man, I’ve got this problem, I’ve got that problem... and now… I can’t afford to buy food. The worrying about it can affect people even more than actually the physical effect of not having it (Shaun Area 3).

### Loss of self-worth & dignity

Linked to mental well-being, many participants described significant impacts on their self-worth and dignity. Participants described a strong sense of disbelief and a profound feeling of failure at needing help from a food bank. This was often compounded by the stigma associated with accessing food banks and the resulting feelings of shame, which over time became internalized and undermined self-worth and personal agency.

Many had found themselves in a situation they never imagined being in, as described by Shazia (Area 1).

It was really difficult… It’s not something I’ve ever used in my whole life before. It’s really hard too… Cause you think that, food bank is when you really, really need help, and I just never thought I'd find myself in that situation... not being able to afford such a basic thing like to feed your kids is really hard.

Many participants also described the emotional struggle of overcoming pride and coming to terms with the need for support from a food bank. This often-included feelings of guilt and the belief that others in more difficult circumstances are more deserving, as illustrated by Peter (Area 3).

I was generally struggling. ...I had no choice but to come into the food banks... it was very stressful, I felt very degraded… because..., I’ve always worked, I never had any handouts…. , it was a very challenging thing for me to get over personally...I was ashamed of myself coming in... These people who were on the streets sort of thing, and then I’m coming in and I’ve got a house, roof over my head, it took a lot for me to try and get through to my mind on this, about coming in to help.

Participants frequently reported feeling embarrassed, with one participant describing that other people seemed to be managing. Such feelings of shame and perception of others caused further emotional distress.

It is an embarrassment, asking for food... I’m just embarrassed and then I have a next-door neighbour and another and they’re coping, you know, and I think oh my God (Wendy Area 3)*.*

### Loss of agency

Many participants described a loss of agency stemming from their inability to provide for themselves and their families. Reliance on external support to survive was particularly difficult when individuals reflected on their previous experiences of independence and autonomy, as highlighted by Joe (Area 1):

It’s shameful that you’ve got to ask for… something you should be able to get for yourself. What the other people you’re asking think of when you’re actually asking for it... Yeah, I’ve worked all my life. That’s why it’s harder to come to this place to be honest. You’ve always been self-sufficient, having [then] to rely on something else.

The lack of control over their lives and household incomes was also a common concern, as described by Zainab (Area 1), who felt that ‘*time is passing by, but you can’t really control your situation, and especially your financial situation getting worse day by day, you know? This really makes you more depressed and stressed; do you know what I mean?*

### Parental guilt and concealed struggles

Those with children commonly described deep feelings of guilt, personal failure, and a sense of failing their children. This created further emotional distress, increasing the stress and anxiety of parents. Zainab (Area 1) describes how this affected her self-worth as a parent:

It makes you feel that you are a failure… You feel that you can’t provide basic needs to your children. So, this is something horrible... not having enough money to buy food, for a mother it’s a big thing. It affects your mental health, ... You try to be strong in front of your children, and then at same time you can’t really hold your emotions back, you can’t really hold your tears back, it is difficult.

Not being able to provide and worrying about the well-being of children deepened parental distress, with many reporting that they often tried to conceal their struggles.

I think just the biggest impact is...not being able to provide for my kids... seeing myself like down there, it makes me bit… How I can call it? I Don't know, like it makes me a bit like upset and angry at the same time, because I don’t know where to go, what to do. (Deborah Area 1)

So, I put a very brave face on, and I don’t let anybody see, especially with my children, what’s going on. I’ve learned if I hide it from them, it’s more better on me. So, I just play the happy person all the time. (Peter Area 3)

### Main findings of this study

Our findings illustrate the profound psychological impacts of food insecurity faced by those accessing food banks. Participants described persistent stress, anxiety, and depressive symptoms linked to financial hardship and the inability to afford sufficient and nutritious food. This reflects recent survey findings from the Food Foundation that reported over two thirds of respondents reported feelings of anxiety and over a half reported increased stress as a result of not having access to reliable food sources.^2^ Food insecurity is inextricably linked with common mental health problems as defined by the Mental Health Foundation 2016,^26^ with worry, anxiety, and stress being common experiences and key indicators of poor mental health.^27–29^ The constant pressure to manage limited budgets and meet household needs left many feeling overwhelmed. Contributing to an increased sense of helplessness and an emotional strain that had detrimental impacts on their mental health. Given the sample was largely unemployed it is likely these impacts were compounded by the fact many participants will have been reliant on state benefits as a source of income. The implications are especially serious given the Cost-of-Living Crisis, which has driven higher rates of food insecurity and hardship among populations across the UK.

Parents in particular reported increased emotional distress due to the inability to adequately provide food for their children. Feelings of guilt and parental failure were common, affecting self-esteem and intensifying poor mental well-being. These findings align with existing literature indicating that food insecurity can exacerbate parental stress and anxiety, diminish self-worth, and lead to detrimental impacts on mental health.^30^^,^^31^ Seeking help from a food bank was a difficult experience, creating an emotional burden. Many participants described feelings of shame and embarrassment, and a perceived loss of dignity from having to rely on external support. These perceptions were further compounded by a sense of not feeling ‘deserving’ of help, believing that others were worse off or coping better, often leading them to question their own capabilities and consequently causing further emotional distress. Such perceptions intensified feelings of shame and made it harder to seek support. It is well documented that stigma and internalized shame can prevent people from accessing food aid, even when they are in need.^32–34^ Stigma and shame can also impact self-worth and agency, as reflected in participant narratives around the shame experienced and reluctance to seek help.^34^

Our findings demonstrate how individuals in food insecurity face internal battles of consciousness and often perceive they are, in many ways, sacrificing their dignity when seeking food support. Regardless of the benefits that food banks may bring, there is an inherent sense of stigma attached to their use and accepting food charity can come with significant costs to their mental health.^35–38^

### What is already known about this topic

There is growing recognition that food insecurity is a driver of poor mental and physical health. Previous studies have shown that individuals experiencing food poverty often report stress, anxiety, and depression. Furthermore, studies report an increased risk of depression for those in food insecurity.^4^^,^^39^ Parents in food-insecure households frequently describe feelings of guilt and inadequacy, and stigma around food bank use can prevent people from accessing support.^22^^,^^32^^,^^33^

### What this study adds

This study provides insights into the lived experiences of food bank users, highlighting the emotional and psychological toll of food insecurity. It documents experiences of those living in the North-West of England, an area with limited research on food insecurity, during an unprecedented time period shaped by the Cost-of-Living Crisis. This study highlights the mental health toll of food insecurity, which is often accompanied by parental guilt and a loss of agency. It contributes to existing qualitative research on food bank users’ experiences and stigma^40–42^ by showing how stigma and shame not only influence the perception of deservingness and help seeking behaviors but also impact mental well-being and how these impacts are often intensified for parents with young children. It also shows how financial hardship and the inability to afford food can lead to stress, anxiety, and depression.

### Limitations

We recognize the study was undertaken in three food banks in the North-West region of England and therefore findings may not be generalizable to other parts of the UK or to other food-insecure groups, particularly individuals not accessing food banks. Furthermore, it must be noted that mental health issues disclosed by the participants are self-reported and are not based on clinical diagnoses. At the same time, some participants may have underreported some experiences because of distrust toward researchers and concerns about poverty-related stigma. Finally, while efforts were made to recruit a diverse sample, the interviewees were predominately white (75%) and largely unemployed (81%).

## Conclusion

Our findings emphasize the significant psychological burdens associated with food insecurity, particularly faced by those accessing food banks. Food bank users reported experiences of persistent anxiety, stress, feelings of depression linked to the financial hardships they faced, and limited access to food of their choice. Parents described increased emotional distress and a sense of guilt due to their circumstances and not being able to provide adequately for their children. Additionally, seeking food support itself was an emotional burden often accompanied by shame, embarrassment, and a perceived loss of dignity, leading some to question their deservingness of such support. Such internalized perceptions of unworthiness and stigma not only intensify psychological distress but may also act as barriers to accessing support. The rising cost of living and increasing financial hardships faced by individuals means many more people may face mental health impacts of food insecurity. It is important to consider how mental health support can be integrated within food aid provision and reducing stigma of service delivery. In addition, it is important to acknowledge that food insecurity does not often occur in isolation. Food insecure individuals can face multiple issues such as chronic health conditions, mental ill health, and physical inactivity. This study demonstrates the need for interventions grounded in evidence and coordinated policy responses—ones that address the broader determinants of health and are implemented through integrated practice-level, local, and national strategies.

## Data Availability

The data underlying this article cannot be shared due to the nature of consent obtained. For further information about the data, please contact the corresponding author.

## Notes

**Koser Khan**, Senior Research Associate

**Natalija Atas**, Research Fellow

**Daniel Clarkson**, Senior Lecturer in Public Health and Social Care

**Linda Heaton**, Honorary Researcher

**Becky Shaw**, Public Advisor

**Adele Thomas**, Public Advisor
